# Thermodynamic analysis of the interactions between human ACE2 and spike RBD of Betacoronaviruses (SARS‐CoV‐1 and SARS‐CoV‐2)

**DOI:** 10.1002/2211-5463.13525

**Published:** 2022-12-09

**Authors:** Agnieszka Rombel‐Bryzek, Adriana Miller, Danuta Witkowska

**Affiliations:** ^1^ Institute of Medical Sciences University of Opole Poland; ^2^ Faculty of Chemistry University of Wroclaw Poland; ^3^ Institute of Health Sciences University of Opole Poland

**Keywords:** binding interactions, human ACE2, isothermal titration alorimetry, receptor‐binding domain, SARS‐CoV‐1, SARS‐CoV‐2

## Abstract

There are many scientific reports on the interaction of the SARS‐CoV‐2 virus S protein (and its RBD) with the human ACE2 receptor protein. However, there are no reliable data on how this interaction differs from the interaction of the receptor binding domain of SARS‐CoV‐1 with ACE2, in terms of binding strength and changes in reaction enthalpy and entropy. Our studies have revealed these differences and the impact of zinc ions on this interaction. Intriguingly, the binding affinity of both RBDs (of SARS‐CoV‐1 and of SARS‐CoV‐2) to the ACE2 receptor protein is almost identical; however, there are some differences in the entropic and enthalpic contributions to these interactions.

## Graphical abstract

We developed a method to obtain reliable data and find differences in binding affinity, binding enthalpy and entropy for the interactions of the RBD proteins of SARS‐CoV‐1 and SARS‐CoV‐2 with the human ACE2 receptor. We also investigated the effects of zinc ions on the RBD of SARS‐CoV‐2 with ACE2 interactions.

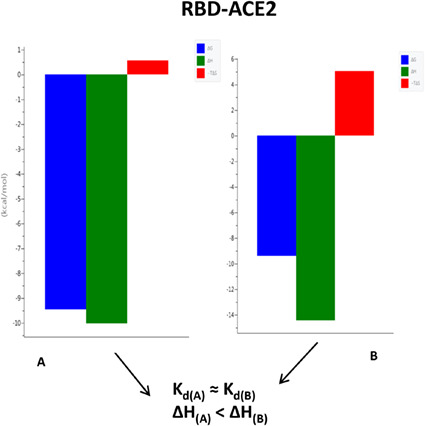

**Abbreviations**
CD: circular dichroism
COVID‐19: coronavirus disease 2019
hACE2: human angiotensin‐converting enzyme 2
HEK: human embryonic kidney
ITC: isothermal titration calorimetry
NTD: N‐terminal domain
RBD: receptor‐binding domain
RBM: receptor‐binding motif
SARS‐CoV‐1: severe acute respiratory syndrome coronavirus‐1
SARS‐CoV‐2: severe acute respiratory syndrome coronavirus 2
SMD: steered molecular dynamics
TRIS: tris(hydroxymethyl) aminomethane

Coronaviruses are enveloped, large, moderately pleomorphic, positive‐stranded RNA (+ssRNA) viruses [1, 2] belonging to the family Coronaviridae [3]. They are divided into four major genera: Alphacoronaviruses, Betacoronaviruses, Gammacoronaviruses, and Deltacoronaviruses [1, 3]. Both severe acute respiratory syndrome coronavirus‐1 (SARS‐CoV‐1) and severe acute respiratory syndrome coronavirus‐2 (SARS‐CoV‐2) belong to the genus Betacoronavirus [1, 3, 4]. SARS‐CoV‐1 (responsible for the SARS epidemic in 2002–2004) and SARS‐CoV‐2 (responsible for COVID‐19) share many similarities. Both viruses cause respiratory diseases transmitted by contact with infected individuals [5]. The genomes of SARS‐CoV‐1 and SARS‐CoV‐2 have 79.5% sequence identity [6] and encode the nonstructural replicase polyprotein, four structural proteins: spike (S), envelope (E), membrane (M), and nucleocapsid (N), and several additional non‐structural proteins called accessory proteins [7, 8, 9].

Membrane proteins, the most abundant proteins in coronaviruses, are responsible for the shape and size of virions [9, 10]. In addition, the M protein is responsible for the processing, modification, and transport of many viral compounds, as well as the formation and release of new virions. The M protein of SARS‐CoV‐2 has 90.5% homology with the M protein of SARS‐CoV‐1 [10]. The envelope protein is multifunctional and participates in virion particle assembly and budding [9]. The nucleocapsid protein enters the host cell with the coronavirus genetic material and enables replication, RNA transcription, assembly and release of the virus [9]. The N protein of SARS‐CoV‐2 is approximately 90% similar to the N protein of SARS‐CoV‐1 [10].

The spike protein (150 kDa) is a highly glycosylated homotrimer [11] that is distributed on the surface of the virion particles and protrudes radially from the viral envelope, forming a “crown‐like” structure [7, 12]. The S‐glycoprotein is a class I fusion protein and is responsible for the binding of the virion to the host receptor, its fusion with it, and entry into the virus [13]. Both SARS‐CoV‐1 and SARS‐CoV‐2 utilize angiotensin‐converting enzyme 2 (ACE2), an enzyme found on the outer surface of a variety of cells, as their cellular receptor [14, 15, 16, 17]. Previous studies have reported that the structure of the SARS‐CoV‐2 S protein is similar to that of the SARS‐CoV‐1 S protein [5, 18, 19, 20].

Each coronavirus spike protein consists of three segments: an ectodomain, a transmembrane anchor, and an intracellular tail [21]. Two subunits can be distinguished in the ectodomain of the S protein [13, 22]. The amino‐terminal subunit (S1) is responsible for the binding of the virus to the ACE2 receptor, while the carboxyl‐terminal subunit (S2) is responsible for the fusion of the virion with the cell membrane, during which it undergoes a conformational change and rejects the S1 subunit [13, 22]. The spike proteins of SARS‐CoV‐1 and SARS‐CoV‐2 differ in length. The S protein of SARS‐CoV‐1 contains 1255 amino acid residues [13], while the S protein of SARS‐CoV‐2 has 1273 amino acid residues [23, 24].

The S1 subunit of coronaviruses includes the N‐terminal domain (NTD), the receptor‐binding domain (RBD), and two subdomains SD1 and SD2 [25], while the S2 subunit contains a fusion peptide and two heptad repeat regions (HR1 and HR2) [13, 22, 25].

A critical first step for SARS‐CoV‐1 and SARS‐CoV‐2 entry into host cells is binding to the ACE2 receptor. A RBD located in the middle region of the S1 subunit specifically recognizes ACE2 and binds to the membrane portion of the receptor's claw‐like structure [4, 6, 19, 24]. The RBD region of SARS‐CoV‐1 and SARS‐CoV‐2 has a sequence identity of approximately 73–76% [4]. The overall structure of the RBD of the two viruses is similar. It contains a core and an extended loop called the receptor‐binding motif (RBM), which interacts directly with ACE2 [19, 24]. The core of the RBD has a five‐stranded antiparallel β‐sheet (β1–β4 and β7) and three short α‐helices (α1–α3), while the RBM is located between the β4 and β7 strands and contains the short β5 and β6 strands [8, 19].

Nevertheless, many findings suggest some internal sequence and structural differences between the RBD of SARS‐CoV‐1 and SARS‐CoV‐2 [19]. The RBD of SARS‐CoV‐1 and SARS‐CoV‐2 spans full‐length amino acid residues 318–510 and 331–524 of the S protein, respectively [8, 10].

Recent structural studies show that extensive interactions occur between RBD and ACE2 [18, 19, 20, 22]. ACE2 is a homodimer, with each monomer containing an N‐terminal peptidase domain, a C‐terminal collectrin‐like domain, a single‐pass transmembrane region, and a short cytoplasmic region. The RBD‐binding region of ACE2 is located in its N‐terminal peptidase domain [20].

Many previous studies reported variations and conformational differences at the interfaces of SARS‐CoV‐1 and SARS‐CoV‐2 with the ACE2 receptor [19, 20, 24].

They showed a higher binding affinity between ACE2 and SARS‐CoV‐2 S protein than the binding affinity between ACE2 and SARS‐CoV‐1 S [3, 18, 26, 27].

This is the point where more sophisticated methods could be used. One of these methods is isothermal titration calorimetry (ITC). Indeed, ITC is the most efficient quantitative method for the determination of thermodynamic properties related to the interactions between two molecules.

This study focuses on the analysis of the differences in ACE2 binding by the S protein SARS‐CoV‐1 and SARS‐CoV‐2. Thanks to a carefully designed methodology, we were able to obtain reliable data on binding affinity, stoichiometry, binding enthalpy, and entropy for these interactions and hope to draw the correct conclusions. Although COVID‐19 is evolving from a pandemic to an endemic disease, Betacoronaviruses can still be dangerous, especially to the elderly and healthcare workers, and it is worthwhile to know as much as possible about their interaction with the human receptor protein [28].

## Materials and methods

### Materials

Buffers, namely PBS and tris(hydroxymethyl)aminomethane hydrochloride (TRIS–HCl) were purchased from Sigma‐Aldrich (St. Louis, MO, USA), NaOH and NaCl were from Chempur (Piekary Śląskie, Poland). All reagents were of analytical grade. Deionized water with a conductivity of no more than 0.06 μS·cm^−‐1^ was used to prepare all aqueous solutions.

**Fig. 1:**
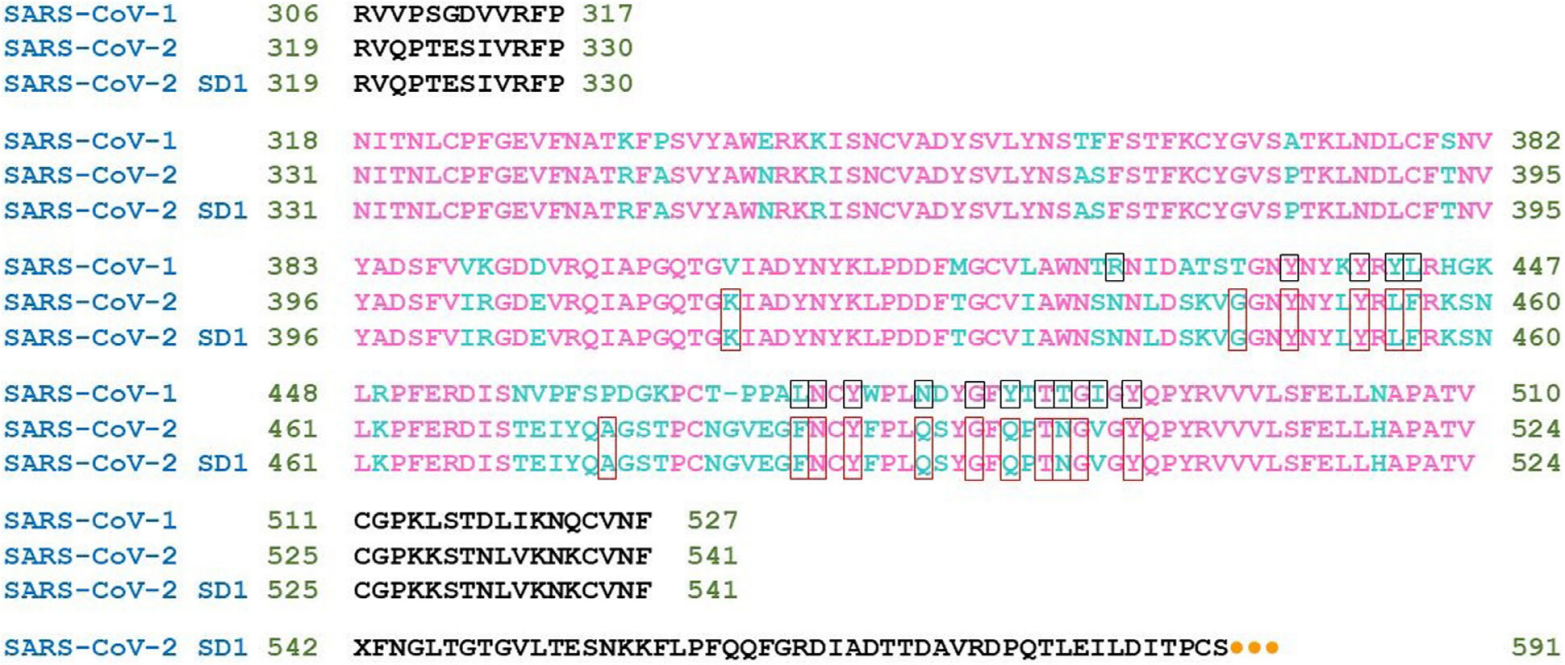
Sequence alignment of SARS‐CoV‐1 S1 RBD, SARS‐CoV‐2 S1 RBD and SARS‐CoV‐2 S1 RBD‐SD1, named within this work: RBD^CoV1^, RBD^CoV2^, and RBD^CoV2b^, respectively. Residues composing RBD are in magenta. Variable amino acid residues between SARS‐CoV‐1 and SARS‐CoV‐2 RBDs are in cyan. The residues of RBD that form bonds with ACE2 are in frames. Orange dots represent monomeric Fc tag.

The SARS‐CoV‐1 spike RBD (RBD^CoV1^) and SARS‐CoV‐2 spike RBD (RBD^CoV2^) proteins were purchased from ABclonal (Woburn, MA, USA) (catalog numbers: RP01299 and RP01258, respectively). Both proteins are His‐tagged and produced in the HEK293 cell expression system. The RBD^CoV1^ consists of Arg306‐Phe527 from SARS‐CoV‐1 spike RBD (Accession; NO_828851.1). The RBD^CoV2^ consists of Arg319‐Phe541 from SARS‐CoV‐2 spike RBD (Accession; YP_009724390.1) (Fig. 1). The proteins hadpurity greater than 95% as determined by SDS/PAGE and by HPLC. The human ACE2 protein (hACE2) was purchased from Elabscience (Wuhan, China) (catalog number: PKSR030508). It is a recombinant, His‐tagged protein consisting of Met1‐Ser740 of human ACE2 (GenBank accession; NP_068576.1) expressed from HEK293 cells. According to the manufacturer, the protein has a purity of over 95% as determined by SDS/PAGE.

The *RBD* ^*CoV2b*^ *and S477D‐RBD* ^*CoV2b*^ proteins were kindly provided by Jason McLellan and Kaci Erwin (Department of Molecular Biosciences, University of Texas, Austin, USA). The plasmid JSM‐1175 used for this expression encodes SARS‐CoV‐2 RBD + subdomain‐1 (residues 319–591) with an N‐terminal artificial signal sequence and a C‐terminal HRV3C protease cleavage site, a monomeric Fc tag, and an 8×His tag. The amino acid sequence is shown in Fig. 1. Detailed information on protein expression and purification can be found in Wrapp et al. [18].

### Dialysis and ITC experiments

Isothermal titration calorimetry measurements were performed at 25 °C and pH 7.4 on a MicroCal PEAQ Isothermal Titration Calorimeter (Malvern Panalytical Ltd., Malvern, UK). After the instrument was stabilized at 25 °C, 40 μL of RBD^CoV1^, RBD^CoV2^ or RBD^CoV2b^ buffered solutions were used to titrate 200 μL of ACE2 buffered solutions (concentration initially approx. 10 times lower than that of RBD) by 19 consecutive injections with an interval of 150 s between each drop and a stirring speed of 750 r.p.m. (each test was repeated a few times). The reference cell was filled with distilled water. Data were fitted using MicroCal PEAQ‐ITC analysis software. An initial injection of 0.4 μL was discarded from each data set to remove the effect of titrant diffusion through the syringe tip during the equilibration process. Thermodynamic parameters (binding affinities, enthalpy and entropy changes) were determined by a combination of nonlinear least‐squares fitting and the selection of an appropriate model describing the binding interaction under investigation [29]. CaCl_2_‐EDTA titration was performed to check the instrument and the results were compared with those obtained for the same samples (test kit) using MicroCal. The heat of dilution was subtracted from each injection [30]. All ITC studies were performed after extensive dialysis of proteins against 1 L of buffer at 5 °C. For each ITC assay, the RBDs and hACE2 receptor protein were dialyzed against the same buffer and during the same time period (the buffer was exchanged 4–5 times every 12 h) to ensure that all samples were as pure as possible and fit into the correct buffer to avoid heat changes due to buffer mismatch. Exhaustive dialysis of all proteins was performed in the same container. After dialysis, the RBDs of SARS‐CoV‐1 and SARS‐CoV‐2 were concentrated by centrifugation. The concentration of each protein was determined by measuring the UV absorbance at 280 nm using NanoDrop One C spectrophotometer (Waltham, MA, USA). The theoretical extinction coefficients (calculated using Expasy ProtParam) were as follows: 169 180 m ^−1^·cm^−1^ for hACE2, and 35 340, 33 850, 91 630 m ^−1^·cm^−1^ for RBD^CoV1^, RBD^CoV2^, RBDCoV2b, respectively.

### Circular dichroism

Circular dichroism (CD) spectra were recorded on a Jasco J‐1500 CDspectrometer (Jasco International Co., Tokyo, Japan) in the range 180–350 nm, using a quartz cuvette with an optical path of 0.1 mm. The 10 μL solutions of RBD WT and S477D‐RBD were prepared from the solutions of these proteins after dialysis against 20 mm TRIS buffer, pH 7.4, 200 mm NaCl.

## Results

### Comparison of the thermodynamics of binding of RBD^CoV1^ and RBD^CoV2^ to the hACE2 receptor

After exhaustive dialysis against PBS buffer, pH 7.4, the interactions of RBD^CoV1^ and RBD^CoV2^ with the hACE2 receptor were investigated using ITC. The one‐side binding model provided the best fitting values for stoichiometry (*N*_ITC_), enthalpy change (Δ*H*_ITC_) and an equilibrium constant (*K*_dITC_). In this part of our studies, hACE2 at a concentration of 6 μm was in the cell and RBDs (RBD^CoV1^ or RBD^CoV2^ at a concentration of 92 or 91 μm, respectively) were in the syringe. The average data of the two best fits are shown in Table 1. Both receptor binding domains (of SARS‐CoV‐1 and SARS‐CoV‐2) bind the hACE2 protein receptor with similar and high affinity and with a stoichiometry (*N*_ITC_) = 1.

**Table 1:** Differences in the hACE2 receptor binding thermodynamics between RBD of SARS‐CoV‐1 and RBD of SARS‐CoV‐2, at pH 7.4 and 25 °C.

	RBD^CoV1^	RBD^CoV2^
*K*_dITC_ (nm)	145.5 ± 25.0	144.0 ± 35.3
Δ*H*_ITC_ (kcal·mol^−1^)	−10.50 ± 0.27	−16.15 ± 0.60
*N*_ITC_	1.03 ± 0.01	0.99 ± 0.02
−*T*_Δ_ *S*_ITC_ (kcal·mol^−1^)	1.15	6.81

**Fig. 2:**
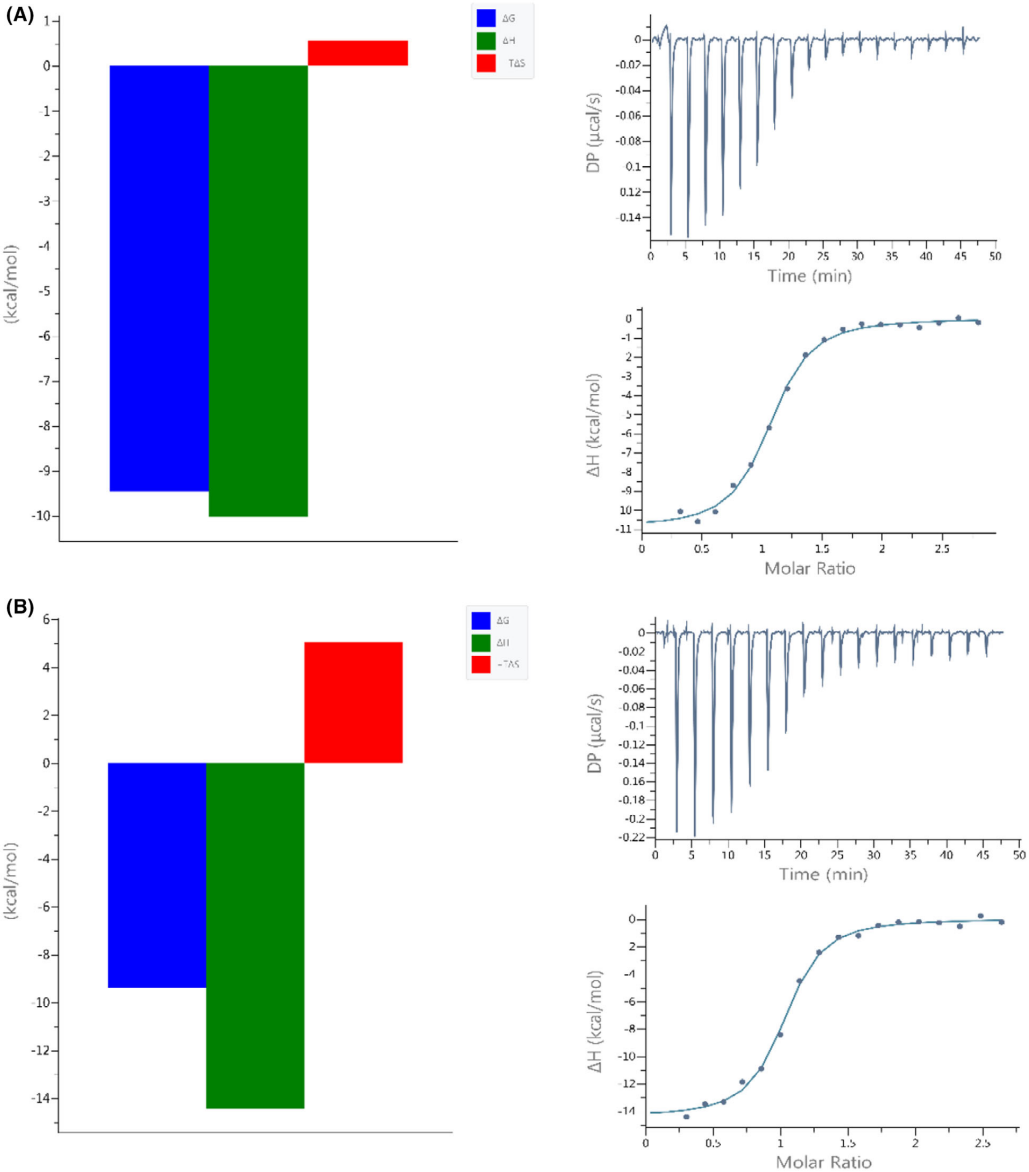
Signatures (left panels) and calorimetric titration isotherms (right panels) of binding of (A) SARS‐CoV‐1 RBD and (B) SARS‐CoV‐2 RBD to the hACE2 receptor protein under the same experimental conditions (PBS buffer, pH 7.4, 25 °C). The concentration of hACE2 was 6 μm and the concentration of the RBDs was in the range of 91–92 μm.

Both systems are enthalpy driven and have very similar Δ*G*_ITC_ (the change in free energy), as shown in Fig. 2. However, the difference can be seen in the Δ*H*_ITC_ and Δ*S*_ITC_ values. When ACE2 interacts with RBD^CoV2^, both the enthalpic contribution (Δ*H*_ITC_) and entropic penalty are larger than when it interacts with RBD^CoV1^ (Table 1). The enthalpy gain of the RBD^CoV2^ interaction is largely compensated by an entropy loss, resulting in no difference in affinity (*K*_dITC_).

**Fig. 3:**
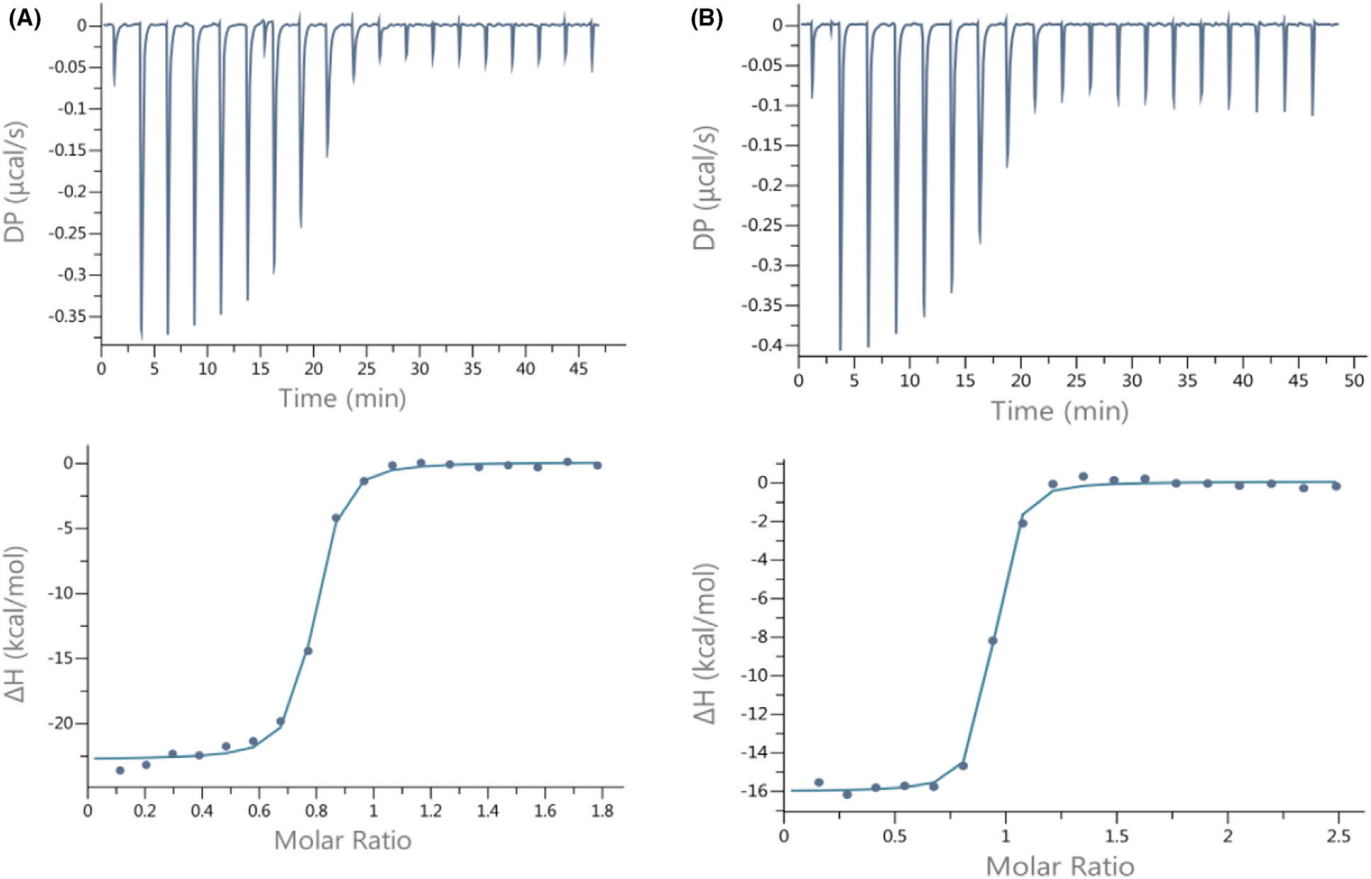
Calorimetric titration isotherms of binding of (A) wild type SARS‐CoV‐2 RBD and (B) SARS‐CoV‐2 RBD mutant (S477D) to the hACE2 receptor protein under the same experimental conditions (TRIS buffer, pH 7.4, 25 °C). The concentration of hACE2 was 9 μm and the concentration of the RBDs was in the range of 86–120 μm.

We hypothesized that the Ser477 residue might be critical for the RBD of SARS‐CoV‐2 and ACE2 interaction. In the Omicron RBD S477 is substituted with N, and that substitution, S477N, has positive impacts on the binding of the Omicron RBD to ACE2 [31]. We were interested if the negatively charged residue (Asp, D) will have opposite impact on RBD to ACE2 binding. The longer fragment of the S protein of SARS‐CoV‐2 (539 amino acid residues in total, including residues 319–591 of the RBD), named by us RBD^CoV2b^, was over‐expressed for this part of our studies, as described in the methodology section. The S477D mutant of RBD^CoV2b^ was constructed and over‐expressed. The RBD^CoV2b^ protein, its S477D mutant and ACE2 were dialyzed against 20 mm TRIS buffer, pH 7.4 + 200 mm NaCl (the buffer was replaced 4–5 times every 12 h). ITC measurements were then performed (Fig. 3) and the results compared. The best‐fit values were obtained by nonlinear least‐squares analysis of the data to the one‐site model. The affinity of the SARS‐CoV‐2 RBD protein and its S477D mutant to the human ACE2 receptor was very similar (*K*_d_ = 20.1 ± 2.73 μm and 20.2 ± 4.22 μm, respectively).

**Fig. 4:**
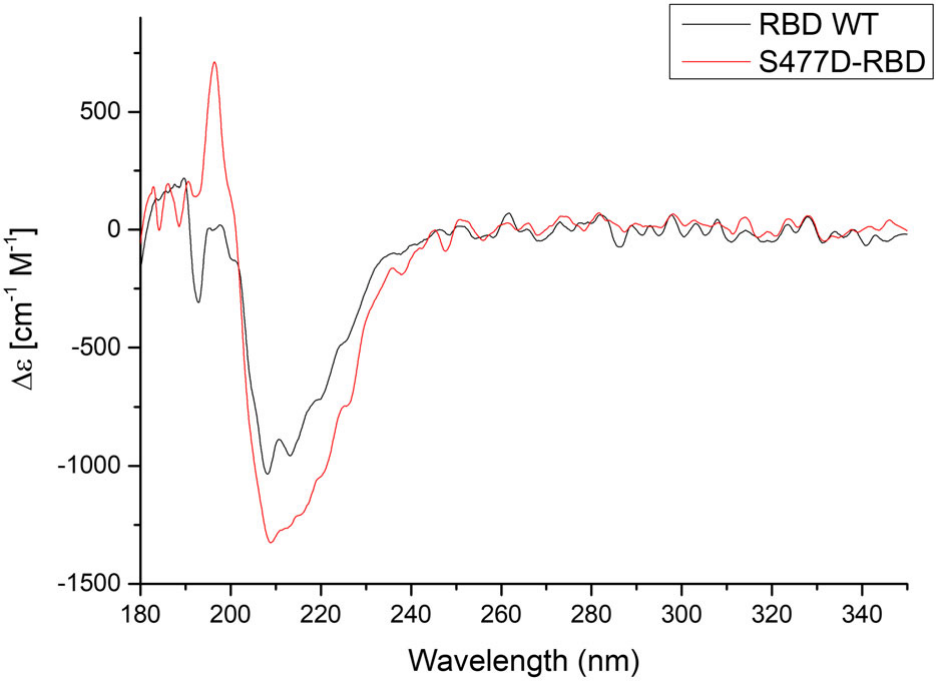
CD spectra of SARS‐CoV‐2 RBD (WT) and single amino acid mutation (S477D) in RBD.

To compare the secondary structures of RBD and its mutant far‐UV CD spectroscopy measurements were performed (Fig. 4). Results indicate, that secondary structures of both proteins are rather comparable, with a slightly higher proportion of the alpha‐helical structure in S447D‐RBD mutant. This observation was also supported by calculations done in k2d3 program.

### The influence of Zn(II) ions on the binding of RBD^CoV2b^ to the ACE2 receptor

Next, we wanted to investigate whether the presence of zinc ions in the buffer has an effect on the binding strength and thermodynamic forces of the RBD‐ACE2 interaction. On the one hand, the ACE2 protein is known to be a zinc metalloenzyme (PBD: 1R42) and the presence of Zn^2+^ in the buffer might also be necessary to maintain the correct ACE2 structure; on the other hand, there are hypotheses that zinc has anticoronaviral properties [32, 33].

**Fig. 5:**
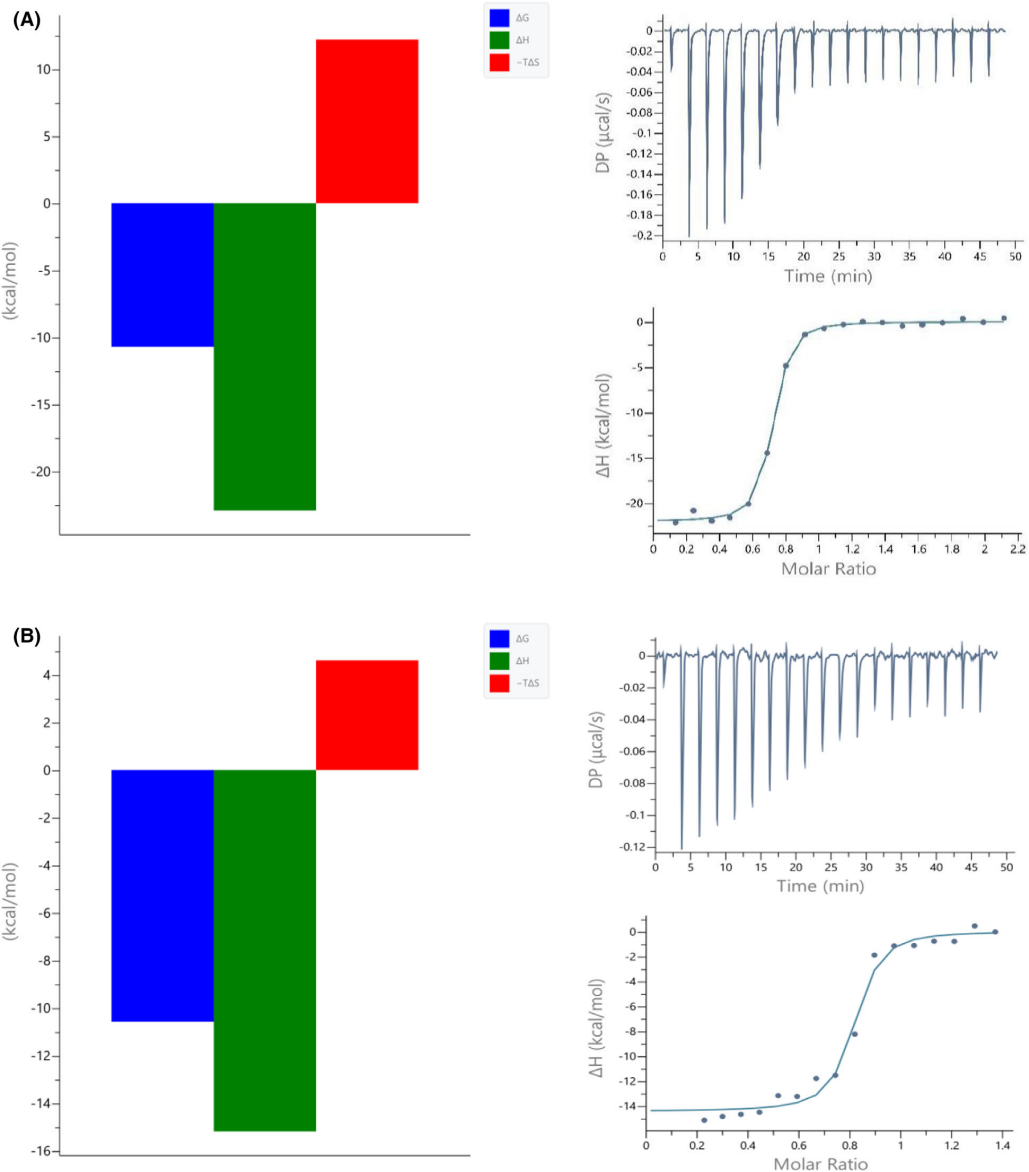
Signatures (left panels) and calorimetric titration isotherms (right panels) of the binding ofRBD^CoV2b^ to the hACE2 receptor in buffer without the addition of ZnCl_2_ (A) and with the addition of 1 mm Zn^2+^ ions (B). The concentration of hACE2 was 4.5 μm and the concentration of the RBD was in the range of 33–55 μm.

Both interactions are enthalpy driven in buffers with and without zinc ions. Large entropic penalties are observed for both systems, especially for the ACE2‐RBD^CoV2b^ interaction studied in the buffer without zinc ions (Table 2 and Fig. 5).

**Table 2:** Binding of the ACE2 protein receptor by RBD of SARS‐CoV‐2, after dialysis of both binding partners in TRIS buffer containing 1 mm Zn^2+^ (left column) and without Zn^2+^ (right column), at pH 7.4 and 25 °C.

	ACE2 – RBD^CoV2b^ (buffer with Zn^2+^ ions)	ACE2 – RBD^CoV2b^ (buffer without Zn^2+^ ions)
*K*_dITC_ (nm)	17.9 ± 9.4	15.9 ± 2.3
Δ*H*_ITC_ (kcal·mol^−1^)	−15.2 ± 0.8	−22.15 ± 0.25
*N*_ITC_	0.79 ± 0.01	0.83 ± 0.04
−*T*_Δ_ *S*_ITC_ (kcal·mol^−1^)	4.61	11.4

## Discussion

### Comparison of the thermodynamics of RBD^CoV1^ and RBD^CoV2^ binding to the hACE2 receptor

There are many reports on the binding strength and conformation of the S1‐RBD protein during interaction with the human ACE2 receptor, for both SARS‐CoV‐1 and SARS‐CoV‐2. However, due to the different techniques used (with variable experimental conditions) and the different S1‐RBD and ACE2 variants studied, it is difficult to compare the binding affinity and other characteristics of ACE2 binding to RBDs of different Betacoronaviruses.

Regardless, the interactions of the S1 protein of SARS‐CoV‐2 and its RBD with the human ACE2 receptor protein have already been studied under variable and not always well‐defined conditions. Reported affinity (*K*_d_) values range widely from 1 to 133 nm [17, 18, 24, 34, 35, 36, 37, 38]. The S1 protein of SARS‐CoV‐1 and its binding to the human ACE2 receptor have also been studied. The *K*_d_ value of these interactions varied from 5 to 325.8 nm [17, 18, 19, 39]. These studies were mainly performed with surface plasmon resonance or biolayer interferometry.

In our study, the interactions were investigated under identical experimental conditions. Even dialysis of all binding partners (RBD^CoV1^, RBD^CoV2^ and ACE2 proteins) was performed simultaneously in the same buffer. In addition, ITC offers a particular advantage when measuring protein–protein interactions in solution and without covalent modification of the proteins. To obtain a complete picture of an interaction, both enthalpic and entropic contributions must be considered, and these were revealed by our studies.

Surprisingly, the RBDs of both viruses bind the ACE2 receptor with very similar affinity (*K*_dITC_ of 145.5 and 144 nm). However, in the case of the RBD of SARS‐CoV‐2, a higher enthalpic contribution (Δ*H*_ITC_ = −16.5) was detected, which was also compensated by a larger entropic disadvantage (−*T*Δ*S*_ITC_ = 6.81) than in the case of the RBD^CoV1^‐ACE2 system (Table 1). The more exothermic Δ*H* could be due to the formation of more energetically favorable non‐covalent interactions between RBD^CoV2^ and ACE2.

**Fig. 6:**
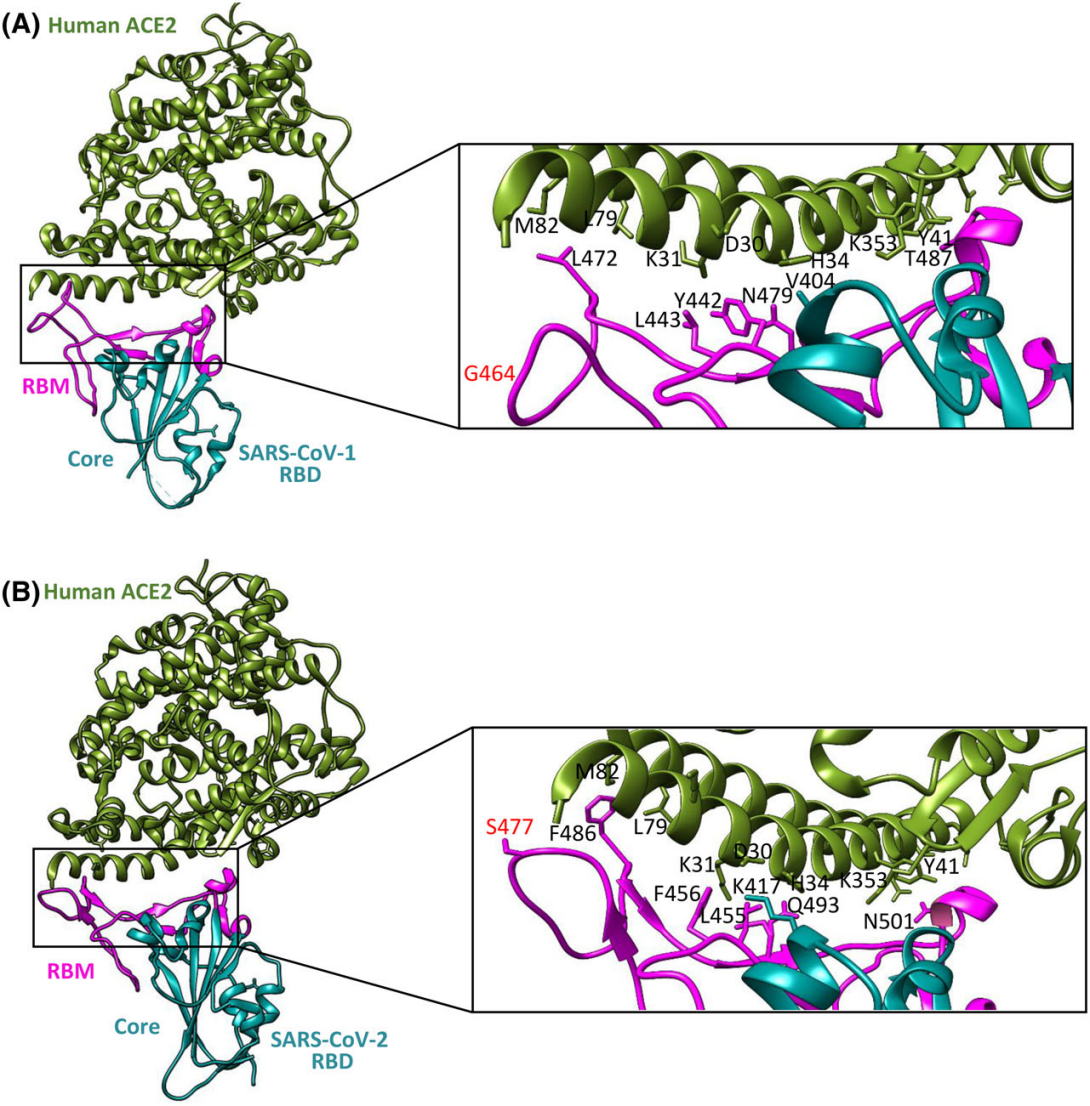
Comparisons of interactions at the SARS‐CoV‐1 RBD‐ACE2 (A) and the SARS‐CoV‐2 RBD‐ACE2 (B) interfaces. Contacting altered residues are shown as sticks and labeled. ACE2 is green, the core of RBD is in cyan, and RBM is in magenta. PDB ID for SARS‐CoV‐2 RBD‐ACE2 is 6M0J; PDB ID for SARS‐CoV‐1 RBD‐ACE2 is 2AJF.Visualized by uscf chimera [44].

The valine residue at position 404 in the S protein of SARS‐CoV‐1 is replaced by a unique residue (Lys417) in the RBD of SARS‐CoV‐2. As has been shown, unlike Val404 in the RBD of SARS‐CoV‐1, Lys417 forms salt bridge interactions with Asp30 of ACE2 (Fig. 6) [19].

However, as shown by Lan et al. [19] there are 13 hydrogen bonds and 2 salt bridges at the interface between SARS‐CoV‐2 and ACE2 compared to 13 hydrogen bonds and 3 salt bridges at the interface between SARS‐CoV‐1 and ACE2. Moreover, the major contact residues between the RBD of SARS‐CoV‐1 and SARS‐CoV‐2 are largely conserved, suggesting that there may be a different reason for the higher energy of the RBD^CoV2^‐ACE2 interaction than that of the RBD^CoV1^‐ACE2 interaction. Recent work on the interactions of the RBD variants of SARS‐CoV‐2 with the ACE2 protein showed that only one of the RBD mutations studied (S477N) exhibited an increased enthalpy change [40]. In the RBD of the S protein of SARS‐CoV‐1, Gly464, which does not bind, corresponds to Ser477 of SARS‐CoV‐2. We hypothesized that the Ser477 residue may be crucial for the RBD^CoV2^‐ACE2 interaction and responsible for the increased apparent enthalpy change. However, additional ITC studies with the S477D mutant showed that this mutation do not impact affinity to bind ACE2. The far‐UV CD spectra of WT RBD and S477D mutant were also similar, what suggest that the mutation do not significantly affect the global structure of RBD.

It is known that sometimes a residue or protein component that does not interact directly with its partner protein can modulate the kinetics and thermodynamics of the molecular recognition process. It has been shown that the region consisting of residues 475–487 is also the key flexible region within the RBM of the SARS‐CoV‐2 S protein [41]. The question arises whether the greater entropic disadvantage is due to this region becoming more ‘rigid’ upon binding of ACE2? It seems likely that this is the case. However, the divergence in the entropy component could be related to the glycan profile of both ACE2 and the RBD of the S protein. The SARS‐CoV‐2 S protein has a different glycan profile than other coronaviruses [11, 42]. Glycans have intrinsic flexibility around glycosidic bonds. For this reason, the loss of conformational entropy of the sugar during protein–protein interaction has thermodynamic consequences that can be captured by ITC. Interestingly, controlled molecular dynamics (SMD) analysis showed that the Asn90 glycan of ACE2 can hinder the association of RBD^CoV2^ with ACE2 more than RBD^CoV1^, but makes the dissociation of RBD^CoV2^‐ACE2 more difficult than that of RBD^CoV1^‐ACE2 [3].

In summary, it appears that RBD of SARS‐CoV‐2 has more optimal interaction points, but this interaction leads to greater order in the RBD^CoV2^‐ACE2 complex than RBD^CoV1^‐ACE2, resulting in a more negative Δ*S*° component.

### The influence of Zn^2+^ ions on the binding of RBD^CoV2b^ to the ACE2 receptor

This part of our studies was designed to examine the influence of Zn^2+^ ions on binding with hACE2. We can compare the changes in affinity, enthalpy and entropy between the interaction of RBD^CoV2b^ with the human receptor ACE2 after dialysis in a buffer containing 1 mm Zn^2+^ and without zinc ions because the conditions are almost identical. The results show that the zinc ions present in the buffer have no positive impact on RBD‐ACE2.

A slightly higher affinity of RBD to ACE2 studied in buffer without Zn (II) ions (15.9 ± 2.3 nm) and the fact that the process is more enthalpically driven (Δ*H* = −22.15 ± 0.25) can be caused by the weak interaction of zinc ions with the TRIS buffer in the second case (Table 2). The Zn(II)‐TRIS interaction was shown to have an affinity of 33 mm [43]. Nonetheless, it is quite reasonable to perform such interaction studies in PBS buffer without the addition of zinc ions, as most researchers do.

We are aware that the affinity and thermodynamic properties of the binding of the ACE2‐S1 protein do not provide all the information about the interaction of the SARS‐CoV‐2 virus with the human receptor. Nevertheless, our results provide a solid basis for future studies on SARS‐CoV‐2 and its mutants. Since the RBM of the S1 protein forms an unstructured loop (Fig. 6), our future perspective is to design a spike protein fragment containing this loop of SARS‐CoV‐2 and its variants to identify the unique effects of S1‐specific mutations on ACE2 binding. The full thermodynamic properties may provide an answer to the questions of which mutations are central to this interaction and why some of the new variants of SARS‐CoV‐2 spread more rapidly.

## Conflict of interest

The authors declare no conflict of interest.

## Author contributions

AR‐B and DW designed the study, conducted the experiments, and wrote the manuscript. AM performed the CD spectroscopy experiments.

## Data Availability

Additional data (e.g., more ITC results) supporting the findings of this study are available from the corresponding author upon request.
